# Editorial: Acute and chronic changes in postural control in response to different physiological states and external environmental conditions

**DOI:** 10.3389/fnhum.2023.1152276

**Published:** 2023-03-06

**Authors:** Urs Granacher, Nejc Sarabon, Jan Babič, Thierry Paillard

**Affiliations:** ^1^Department of Sport and Sport Science, Exercise and Human Movement Science, University of Freiburg, Freiburg, Germany; ^2^Faculty of Health Sciences, University of Primorska, Izola, Slovenia; ^3^Laboratory for Neuromechanics, and Biorobotics, Jožef Stefan Institute, Ljubljana, Slovenia; ^4^Faculty of Electrical Engineering, University of Ljubljana, Ljubljana, Slovenia; ^5^Université de Pau et des Pays de l'Adour, E2S UPPA, MEPS, Tarbes, France

**Keywords:** balance, exercise, stress, fatigue, plasticity, patient, athlete

## Introduction

Human postural control is fundamental for motor skill learning and the performance of everyday and sports-related tasks (Gebel et al., 2020). Previously, postural control has been defined as the control of the body's position in space for stability and orientation to maintain and recover balance (Shumway-Cook and Woollacott, 2012). Postural balance is regulated through the complex processing of proprioceptive, vestibular and visual information on a spinal and/or supraspinal level within the central nervous system (Taube et al., 2008). Depending on the postural parameter under investigation, static and dynamic balance follow a *U*-shaped (postural sway) or inverse *U*-shaped curve (gait speed) across the lifespan. In other words, postural balance is not yet fully developed during childhood and adolescence which is for instance established in a larger dependency on visual information for the maintenance of upright stance (Granacher et al., 2011). In seniors, postural balance deteriorates due to biological aging of the central nervous system (e.g., desensitization of muscle spindles) and increased physical inactivity. When confronted with environmental stressors such as bad lightening, unstable surfaces, multitask situations, postural balance is impaired resulting in an increased risk of sustaining injuries and/or falls. Physical exercise or training induce sensations of fatigue resulting in acute balance declines (Gebel et al.). This is indicated for instance in an increased risk of sustaining injuries in the second half of soccer games (Hawkins and Fuller, 1999). While physical exercise can provoke acute negative effects on postural balance (Paillard, 2012), it has also positive effects when chronically applied over longer intervention periods (Paillard, 2017), irrespective of the population under investigation [youth, adult (athletes), seniors]. There is evidence from original research (Granacher et al., 2010a,b; Wälchli et al., 2018) and systematic reviews and meta-analyses (Lesinski et al., 2015a,b; Sherrington et al., 2017; Gebel et al., 2018; Al Attar et al., 2022) on the effects and dose response relations of balance-related interventions (balance training, slackline training, perturbation training etc.) on measures of balance and the risk of injuries and falls. Less is known on the underlying physiological mechanisms responsible for the exercise induced adaptations. With this Frontiers in Neuroscience Research Topic, we encouraged researchers to further explore the topic by providing knowledge on the acute and chronic changes in postural control in response to different physiological states and external environmental conditions to better understand how the human body responds to physiological stress/exercise in terms of postural control.

## Summary of selected articles from this Research Topic

Overall, twelve articles (ten original research articles, one review article, one brief report) were published in this Research Topic between 2021 and 2023. Fifty-five researchers from different countries across the globe including Austria, Canada, China, France, Germany, Hungary, Iran, Italy, Slovakia, Slovenia, South Africa, the Netherlands, and the United Kingdom participated in this Research Topic. In January 2023, the Research Topic received 22,935 total views and 3,496 total downloads. In terms of demographics, the Research Topic received particular interest in the United States of America (2,661 views), Belgium (1,190 views), Germany (504 views), China (254 views), and finally Slovenia (22 views). The twelve articles touched clinical (limb asymmetries, spasticity) and sports-related topics (fatigue, blood flow restriction, compression garments) surrounding the overarching topic postural control. In the clinical setting, the examined study cohorts consisted of obese, post-stroke or knee osteoarthritis patients. In the sports-related setting, researchers examined (youth) athletes as well as the general youth population and healthy adult individuals.

### Fadillioglu et al. (2022)

Although the effects of the stomatognathic motor system on postural balance have been established under static conditions, it has not yet been studied during dynamic steady-state balance. Fadillioglu et al. analyzed the effects of controlled stomatognathic motor activity on the control of the center of mass (COM) during dynamic balance under different stomatognathic motor conditions. The experimental conditions comprised jaw clenching, tongue pressure, and habitual stomatognathic behavior. Findings from this study indicate that deliberate jaw clenching or tongue pressing does not appear to affect dynamic steady-state balance. Due to balance task-specificity, further research is needed on the effects of stomatognathic motor activities on dynamic balance in different movement tasks.

### Fu et al. (2021)

The aim of the study reported in the article of Fu et al. was to compare individuals with knee osteoarthritis and asymptomatic controls with regards to their postural balance and identify kinematic and lower extremity muscle activity characteristics during a stand-to-sit task. A comprehensive experimental study showed significantly larger COM displacements and peak instantaneous COM velocity in the anterior-posterior direction, reduced ankle dorsiflexion range of motion, greater anterior pelvic tilt range of motion, and lower quadriceps femoris and muscles activation level coupled with higher biceps femoris muscle activation level during the stand-to-sit task of the osteoarthritis patients. Findings from this study imply that rehabilitation programs targeting disease-related impairments could be beneficial for restoring the functional transfer in individuals with knee osteoarthritis.

### Gebel et al. (2022)

In this original research article, Gebel et al. aimed to examine the effects of physical and mental fatigue on postural sway and cortical activity in healthy young adults. Before and after balance testing using a pressure sensitive mat and electroencephalography, participants performed in random order an all-out repeated sit-to-stand task to induce physical fatigue and a computer-based attention network test to provoke mental fatigue. The applied physical fatigue protocol resulted in increased postural sway accompanied by enhanced alpha-2 power in the parietal region of interest. Mental fatigue led to increased postural sway variability and alpha-2 power. The observed fatigue-related changes in cortical activity indicate impairments in sensory information processing related to movement planning and execution within the somatosensory cortex, resulting in balance declines.

### Heil (2022)

Inter-limb asymmetries are associated with non-contact injuries, particularly in team sports (Dos'Santos et al., 2021). In this original research article, Heil investigated the influence of different physical fatigue protocols on inter-limb asymmetries during the performance of a dynamic postural control test in a rather large cohort of 128 young and physically active male and female adults. Following a between-subject design, participants were allocated to a fatigue protocol either executed on a bike ergometer (30 s all-out Wingate test) or a treadmill (10 s all-out test at maximal running velocity and a slope of 7.5%). Before and after the fatigue protocol, all participants performed the lower limbs Y-balance test in anterior direction. The analysis revealed that inter-limb asymmetries did not increase due to fatigue. No significant differences were found between the running and the cycling protocol. The author suggested that fatigue protocols of longer duration and acyclic character (e.g., change-of-direction tasks) should be examined in future studies.

### Mahmoudzadeh et al. (2021)

The article of Mahmoudzadeh et al. evaluates the effects of spasticity of ankle plantar flexors on balance in post-stroke patients and determines the relationship between the spasticity with ankle proprioception, passive ankle dorsiflexion range of motion, and balance confidence. The results of the study show that joint proprioception was significantly better in the low spasticity group compared to the high spasticity group and that there were no significant relationships between the spasticity severity and passive ankle dorsiflexion range of motion, and balance confidence. The authors conclude that postural balance is significantly affected in post-stroke patients, regardless of the severity of the ankle plantar flexor spasticity.

### Noé et al. (2022)

Based on the observation that inter-limb balance asymmetries increase the risk of injury in athletes, Noé et al. analyzed the effects of wearing compression garments (CG) to reduce these asymmetries. Their results show that inter-limb asymmetries were lower with CG in participants with high levels of asymmetries at baseline while they were higher in participants with low levels of asymmetries at baseline. Thus, in order to reduce inter-limb asymmetries and the associated injury risk in athletes, wearing CG has a beneficial effect in the presence of high levels of inter-limb balance asymmetries at baseline. Conversely, CG should be avoided in individuals with low baseline balance asymmetries as they likely produce confusion and overload in sensorimotor processing.

### Russell et al. (2022)

Russell et al. conducted a study to investigate the effects of placebo and nocebo effects on postural stability. While the placebo effect has been shown to impact postural stability before (Villa-Sánchez et al., 2019), Russell et al. included both objective (measured) and subjective (perceived) measures of postural stability. The participants in the placebo/nocebo groups were given an inert capsule described as a potent supplement which would either positively or negatively influence their postural stability. As expected, an increase in body sway and reduced perceived stability were noted in the nocebo condition, and the opposite was true for the placebo group. Additional analyses also revealed that performance expectations heavily influenced the perception of postural instability. These results indicate that postural control (and its perception) is susceptible to expectation manipulation (both placebo and nocebo), which has important practical implications.

### Sozzi and Schieppati (2022)

Sozzi and Schieppati investigated the adaptation of postural control (postural sway) on a compliant surface (i.e., a foam) across eight repetitions, and analyzed how visual sensory information and light touch influence postural control. Previous studies have examined balance adaptations in paradigms with external perturbations. However, balance adaptations during quiet unperturbed stance are unresolved. The authors confirmed that postural sway adaptations occur during standing on a compliant surface. This adaptation is reflected in a progressive increase in the amplitude of the lowest frequencies of the spectrum and a concurrent decrease in the high-frequency range. The authors concluded that the control of balance was shifted from the lower to the higher levels of the nervous system. In addition, the adaptation rate was modestly influenced by light touch.

### Voglar et al. (2022)

In their original research article, Voglar et al. compared the effects of supported and unsupported intermittent trunk flexion on postural control during sitting in healthy adult males and females aged 23–24 years. Previous studies have indicated that prolonged spinal flexion (as in, for example, crane operator work) induces changes in trunk motor control and spinal stiffness (Voglar et al., 2016). However, this was one of the first studies to examine the changes in sitting postural control after prolonged flexion. The results indicated that prolonged intermittent flexion does not induce any changes in center of pressure motion during a seated balance task, regardless of the presence of a trunk support. The authors suggested that this was due to a successful compensation of decreased passive stiffness by increased reflex activity.

### Wiesinger et al. (2022)

In their study, Wiesinger et al. contrasted measures of postural sway in young overweight/obese (YO) and young normal-weight (YN) children and adolescents. Their results indicated postural control deficits in YO compared to their YN peers, reflected in a more rigid postural control. The authors concluded that without targeted balance exercise, YO are susceptible to end up in a vicious circle of poor balance control and low physical activity. The researchers urge practitioners to implement postural balance training in the early years to avoid impairments later in life.

### Willberg et al. (2021)

Muscle fatigue may have acute negative effects on static and/or dynamic postural control which could impair sports performance such as speed skating and alpine skiing. In a within subject design, Willberg et al. examined the acute effects of lower-body blood flow restriction using cuffs around the thighs on static and dynamic postural control in 20 physically active healthy males and females aged 26 years. Using block randomization, participants performed static and dynamic balance tests on a fixed and moveable platform (medio-lateral and antero-posterior direction) in a squatted bipedal position at a knee angle of 110° with inflated cuffs and without cuffs until exhaustion. Blood flow restriction resulted in significantly lower oxygenation of the m. quadriceps femoris and was associated with a significantly lower time to exhaustion compared to the non-restricted condition. Relative postural sway did not differ significantly between non-restricted and the blood flow restriction conditions. In conclusion, blood flow restriction resulted in deoxygenation and a reduced time to exhaustion in the squatted position. Postural control and the ability to regain stability after perturbation were unaffected.

### Zemková and Kováčiková (2023)

Zemková and Kováčiková provide a scoping review article examining sport-specific training-induced adaptations of postural control and their relationship to measures of sport performance. The authors show that there is a relationship between measures of static and/or dynamic balance and sports performance in many sports. This can be attributed to an enhanced ability of athletes to make postural adjustments in highly demanding postural tasks. However, the extent to which sport-specific exercises contribute to their superior postural stability is unknown. The authors concluded that further research is needed to investigate the relative contributions of each of these balance exercises to improve sports-performance.

## Conclusions

This Frontiers Research Topic contributes to our understanding of the acute and chronic changes in postural control due to different physiological states and external environmental conditions in different cohorts such as patients, healthy youth and adults. Information from this Research Topic can be used to design balance training programs for performance enhancement, rehabilitation, and injury prevention. More research is need on the underlying physiological mechanisms responsible for acute and chronic adaptations within the postural control system.

## Author contributions

All authors listed have made a substantial, direct, and intellectual contribution to the work and approved it for publication.
